# Assistive robotic systems in nursing care: a scoping review

**DOI:** 10.1186/s12912-023-01230-y

**Published:** 2023-03-18

**Authors:** Christoph Ohneberg, Nicole Stöbich, Angelika Warmbein, Ivanka Rathgeber, Amrei Christin Mehler-Klamt, Uli Fischer, Inge Eberl

**Affiliations:** 1 Professorship of Nursing Science, Faculty of Social Work, Catholic University of Eichstätt-Ingolstadt, Kapuzinergasse 2, 85072 Eichstätt, Germany; 2 Clinical Nursing Research and Quality Management Unit, University Hospital LMU Munich, Marchioninistr. 15, 81377 Munich, Germany

**Keywords:** Nursing robotics, Assistive robotic systems, Robotic applications, Technology implementation, Inpatient care, Outpatient care, Long term care, Nursing science, Scoping review

## Abstract

**Background:**

The use of assistive robotic systems in care is intended to relieve nursing staff. Differentiated and literature-based findings on current application possibilities, technological developments and empirical findings are necessary to enable a goal-oriented and participatory development of assistive robotic systems of care. The aim of this review was to identify assistive robotic systems and their areas of application in nursing settings. Furthermore, an overview of existing social and nursing science findings from the research field of assistive robotic systems will be described.

**Methods:**

A systematic literature search was performed based on the JBI scoping review methodology. During the period from May to August 2020, the databases MEDLINE via PubMed, CINAHL, Cochrane Library, Web of Science, and IEEE Xplore Digital Library were searched. In order to reflect current developments and evidence in the present literature work, a supplementary search with these same requirements was conducted in January 2022.

**Results:**

The 47 included publications are divided into 15 studies, 23 technical articles and nine opinion-based articles (text-opinion). A total of 39 different assistive robotic systems were identified. There were 55% in the testing phase and 29% of the systems in the development phase. Assistive robotic systems can be divided into six fields of application: Information and patient data processing, assistance with activities of daily living, fetch and bring activities, telepresence and communication, monitoring, safety and navigation, and complex assistance systems. The description of the study findings is divided into "integration of technology and impact on practice" and "attitude and acceptance of elderly people towards assistive robotic systems".

**Conclusion:**

The results of the research show that the use of assistive robotic systems in care mainly take place in the context of development and testing phases. In addition to usability and acceptance issues, implementation factors must be integrated into theory-driven research projects.

## Background

Under the conditions of demographic change and epidemiological developments, Western society is challenged to provide high-quality care that can be financed in the long term [1]. Under this demand, the profession of nursing is confronted with different challenges, such as the shortage of skilled personnel. This has a direct impact on the profession. Hendrich et al. [2] state that nurses spend a large part of their working time on activities that are not related to the patient, such as performing service activities or administrative tasks, which in some cases also results in unnecessary walking. In addition, nursing tasks are frequently interrupted. This is caused by requests from colleagues and patients as well as alarms and telephone calls. The number of interruptions increases with the number of persons to be cared for [2]. These interruptions are additional stressors and reduce the quality of nursing actions as well as the effectiveness of work processes, which can lead to an additional workload in the daily work routine [3, 4]. To counteract these challenges, innovative digital and robotic systems are increasingly entering the nursing sector. Innovative technologies are intended to support nursing staff and contribute to improving the quality of care. One approach here is aimed at supporting people in need of assistance as well as formal and informal caregivers through autonomous robotic systems. The classification of these systems is based less on their technical capabilities. Rather, they are subdivided in terms of their practical use and functions, such as assistance in a social care context, monitoring function, or nursing assistance [5–7]. Another differentiation approach is the subdivision into service robotics and social-assistive robotics. Social-assistive robotic systems can be differentiated into emotional care and cognitive support functions. Systems with cognitive support functions provide mental stimuli through often simple memory exercises [8]. The term service robotics covers technical systems that support humans in performing services and work in a partially or fully automated manner. They are used in non-industrial fields of application and operated by persons who are not specially trained. In addition to informational and sensory functions, service robotics are also capable of locomotion and/or performing complex tasks consisting of multiple steps and materials [9, 10].

Innovative technologies for caregiving are generally hoped to relieve the burden on formal and informal caregivers. However, the development of these technologies is still in its infancy, highlighting the need for fundamental research [11–13]. Differentiated and literature-based findings on current application possibilities, technological developments and empirical findings are necessary to enable a goal-oriented and participatory development of assistive robotic systems for care.

Regarding the state of research, a first orienting search identified reviews that dealt with the topic of robotic systems in nursing with corresponding foci. In the scoping review by Buhtz et al. [14], the possibilities and applications of robotic systems were investigated with a focus on the nursing care context in the home environment. The concept of need for care as described in German social legislation was chosen as the nursing relevance criterion. Maalouf et al. [8] focused in their scoping review on the categorization of robotic systems used in nursing care. Other reviews have referred to specific user groups such as the elderly or people with cognitive impairments [15, 16]. Reviews on assistive robotic systems that consider both cross-sectoral and potential users in care contexts could not be located in an orienting search. This review therefore takes a scientific, practical and user-oriented perspective. In addition to assistive robotic systems, the focus is on nursing fields of action and persons within the nursing and care process.

The aim of this review was to identify assistive robotic systems and their application areas and possibilities in nursing settings, as well as potential users. Furthermore, an overview of existing social and nursing science findings from the research field of assistive robotic systems will be described. For this purpose, the following research questions were guiding:

- For which fields of activity and application are assistive robotic systems being developed in nursing settings?
- What social and nursing science findings are available in the field of assistive robotic systems?

## Method

A systematic literature search was conducted using the PRISMA statement for scoping reviews. For this, the recommendations of the JBI were methodologically followed. This form of systematic literature review was chosen to provide an orientation to the current state of the research literature, to delineate areas of work and topics, and to map key concepts in the research and development field of assistive robotic systems in nursing [17, 18]. The conduct of the review was planned and recorded using the recommendations of Nordhausen and Hirt [19].

### Inclusion and exclusion criteria

The PCC scheme (Population, Concept, and Context) was used to define and differentiate the search components [17]:

- Population: in addition to caregivers, individuals in need of care and relatives were included as users. This heterogeneous composition was chosen in order to be able to make a differentiated statement about the target groups in the field of assistive robotic systems for care.
- Concept: For this review, the term assistive robotic systems was used according to Schraft & Volz [9] and Graf et al. [10] defined. These define assistive robotic systems as partially and fully autonomous robotic systems for non-industrial fields of work, which can be operated by persons who are not specially trained, such as persons in need of care and/or their relatives. In addition, the included assistive robotic systems had to be able to move around and perform complex tasks consisting of multiple steps and/or materials [9, 10]. Systems that affect human cognitive and emotional well-being were excluded. Furthermore, robotic systems for surgery, rehabilitation, and mobilization were excluded.
- Context: In order to comply with the sensitive search principle, the context of the literature search refers not only to the acute inpatient sector, but also to the outpatient and inpatient care sector.

### Search strategy

Based on the methodological guidance for scoping reviews (JBI methodology) [17], the search strategy was iterative and as comprehensive as possible. In addition to primary studies and reviews, gray literature (expert opinions, reports, guidelines) and technology-related articles were included. No restriction was placed on the time period of publication to include early or recent developments in the field. Publications in German and English were considered.

In the first step of the literature search, a general search on the topic of assistive robotic systems for nursing care was conducted in the databases CINAHL and MEDLINE via PubMed. The articles found were analyzed with regard to relevant keywords and key terms (keywords) in the title and abstract. On this basis, the search strategy (Table 1) for the second step of the literature search was developed. The search terms in the search strategy also break down based on the PCC scheme [17]. Within each topic block, the search terms were linked using the OR operator. The three topic blocks were linked using the AND operator. Individual search terms were supplemented with wildcards. In order to obtain more hits, the search terms within the respective databases were extended to the entire text. The second database search was performed in the following databases: MEDLINE via PubMed, CINAHL, Cochrane Library, Web of Science, and IEEE Xplore Digital Library. Within each database, the fields title, abstract, keywords, and full text were searched. The specified search terms given in Table 1 were used identically on all databases. The documentation of the database specific search strings were documented on Excel. The search was supplemented by a free web search via Google Scholar, ResearchGate, and Springerlink. Due to the high number of hits, the reference lists of the individual studies could not be checked. The literature search was conducted in the period from May to August 2020. In order to reflect current developments and evidence in the present literature research, a supplementary search with these same requirements was conducted in January 2022.

**Table 1:** Tabular representation of the search strategy (own representation)

**Search terms of the first database search**
**Population:** Nursing OR Nurse OR Caregivers
**Concept:** Robotics OR Service Robots OR Assistive Robots
**Context:** hospitalized OR inpatient
**Specified search terms for database research**
**Population:** nurse* OR caregiver* OR patients OR patient relatives OR service assistants
**Concept:** robot* OR robotic systems OR assistive robot* OR service robot* NOT surgery
**Context:** hospital OR inpatients OR hospitalized OR acute care OR home nursing OR home health care OR long term care OR acute care setting OR outpatient care OR outreach care
**Search strategy of the second database search**
**PubMed:** "((((((Nurse*) OR (caregiver*)) OR (patients)) OR (""patient relatives"")) OR (""service assistants"") AND ((humans[Filter]) AND (english[Filter] OR german[Filter]))) AND (((((""robot*"") OR (""assistive robot*"")) OR (""service robot*"")) OR (""robotic systems"") AND ((humans[Filter]) AND (english[Filter] OR german[Filter]))) NOT (surgery) AND ((humans[Filter]) AND (english[Filter] OR german[Filter])))) AND ((((((((((""hospital"") OR (""inpatients"")) OR (""hospitalized"")) OR (""acute care"")) OR (""acute care setting"")) OR (""home nursing"")) OR (""home health care"")) OR (""long term care"")) OR (""outpatient care"")) OR (""outreach care"") AND ((humans[Filter]) AND (english[Filter] OR german[Filter])))"
**CINAHL: S1** TX nurse* OR TX caregiver* OR TX patients OR TX "patient relatives" OR TX "service assistants"; **S2** TX robot* OR TX "assistive robot*" OR TX "service robot*" OR TX "robotic systems" NOT “surgery”; **S3** TX hospital OR TX inpatients OR TX hospitalized OR TX "acute care" OR TX "acute care setting" OR TX "home nursing" OR TX "home health care" OR TX "long term care" OR TX "outpatient care" OR TX "outreach care" → S1 AND S2 AND S3
**COCHRANE: #1** = (nurse*) OR (caregiver*) OR (patients) OR ("patient relatives") OR ("service assistant"); **#2** = (robot*) OR ("assistive robot*") OR ("service robot*") OR ("robotic systems") NOT ("surgery); **#3** = ("home nursing") OR ("home health care") OR ("long term care") OR ("outpatient care") OR ("outreach care")) OR ((hospital) OR (inpatients) OR (hospitalized) OR ("acute care") OR ("acute care setting")) → #1 AND #2 AND #3
**Web of Sciene:** (TS = (robot* OR assistive robot* OR service robot* OR robotic systems) AND TS = (nurse* OR caregiver* OR patients OR patient relatives OR service assistants) AND TS = (hospital OR inpatients OR hospitalized OR acute care OR acute care setting OR home nursing OR home health care OR long term care OR outpatient care OR outreach care) NOT ALL = surgery) AND LANGUAGE: (English OR German)
**IEEE Xplore:** ((((((((All Metadata:robot*) OR All Metadata:"service robot*") OR All Metadata:"robotic systems") OR All Metadata:"assistive robot*") NOT All Metadata:surgery))) AND ((((((All Metadata:nurse*) OR All Metadata:caregiver*) OR All Metadata:patient*) OR All Metadata:"patient relatives") OR All Metadata:"service assistants"))) AND (((((((((((All Metadata:hospital) OR All Metadata:inpatients) OR All Metadata:hospitalized) OR All Metadata:"acute care") OR All Metadata:"acute care setting") OR All Metadata:"home nursing") OR All Metadata:"home health care") OR All Metadata:"long term care") OR All Metadata:"outpatient care") OR All Metadata:"outreach care"))

### Selection process

After duplicates were excluded, double-blinded title abstract screening and full-text screening were performed by two independent reviewers (OC, SN). For this purpose, the support tool Rayyan QCRI web app from Qatar Computing Research Institute was used [20]. Articles were selected based on the established inclusion criteria. In case of disagreement, consensus was attempted to be reached via discussion; if this was not possible, a third expert was consulted (EI, FU, WA, RI).

### Data extraction

The following criteria were established in advance for data extraction. First, publication-related data were extracted: Author, publication year, title, country, and publication type. The assistive robotic systems and their possible applications were documented using the following criteria:

- State of technological development: a distinction was made here between development, test phase (testing in the laboratory or in the field), and use in practice.
- Setting: Based on the PCC scheme, it was documented in which nursing setting (acute inpatient, outpatient, inpatient long-term care) the assistive robotic system was used or will be used.
- User: Based on the PCC scheme, it was documented who is the direct user of the assistive robotic system: nurse, person in need of care, relatives, other professional groups.
- Application scenario: The application possibilities and fields of activity of the assistive robotic systems were summarized descriptively. Furthermore, it was noted how the interaction between user and robotic system is defined (e.g. via voice input or tablet).
- Descriptive of application opportunities & research findings: The data were tabulated in a word document. The publication related data of the hits were recorded in the form of an Excel spreadsheet. The extracted data of the application areas were categorized for better overview. The categories were formed inductively using content structuring qualitative content analysis following Kuckartz [21].

## Results

**Fig. 1:**
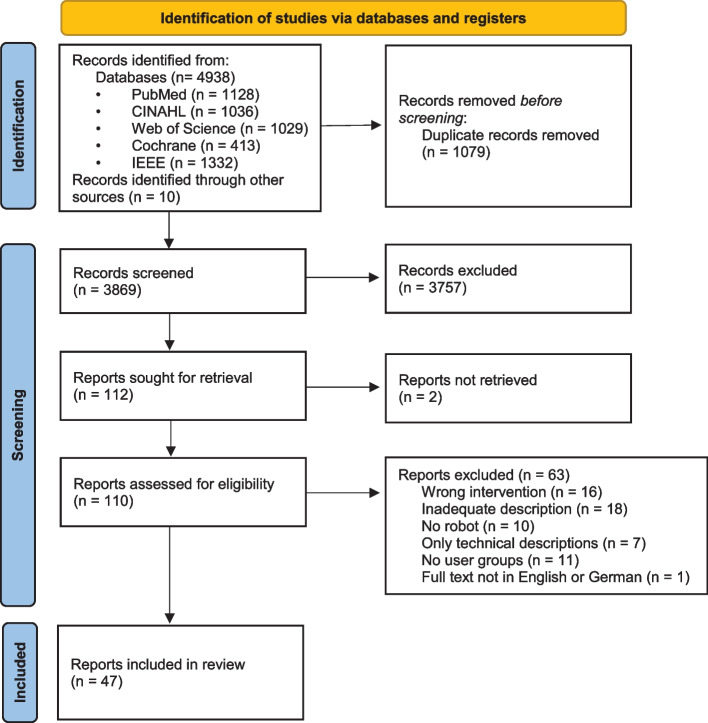
PRISMA flow diagram of the search and selection process (own representation after Page et al. [22])

A total of 4,938 publications were included via database search and an additional 10 via hand search. After excluding duplicates, a total of 3,869 articles were subjected to title abstract screening. Of these, 112 qualified for full text screening. In the end, 47 articles were included in the analysis (Fig. 1).

### Publication characteristics

The 47 included publications are divided into 15 studies, 23 technical articles and nine opinion-based articles (text-opinion). The 15 studies include two systematic reviews (scoping review, systematic review), four qualitative and four quantitative studies, and five studies with a mixed-method approach. The 23 technology articles are divided into 16 conference papers, seven journal articles, and one online publication. The nine opinion-based contributions are divided into four journal articles, two edited volumes, one monograph, one conference paper, and one final project brochure.The publications originate from 25 different countries and were published between 1998 and 2022.

### Overview of assistive robotic systems in nursing care

A total of 39 different assistive robotic systems were identified. Some of the robotic systems were developed for more than one setting or user group. Furthermore, there were the same systems described in different publications in different development phases. There were 55% in the testing phase and 29% of the systems in the development phase. For the clinical setting 56% of the assistive robotic systems were developed. At 48%, just under half were developed for persons in need of care. A differentiated breakdown is shown in Table 2.

**Table 2:** Tabular representation of the robotic systems with regard to state of development, setting and user (own representation)

**State of development**	**Number of robotic systems (*n* = 45)**	**in percent %**
Test phase	n = 25	55%
* Laboratory test*	*n* = *11*	*24%*
* Field test*	*n* = *14*	*31%*
Development phase	n = 13	29%
Use in Practice	n = 7	16%
**Setting**	**Number of robotic systems (*n* = 43)**	**in percent %**
Clinical care	n = 24	56%
Home care	n = 11	25%
Inpatient long-term care	n = 8	19%
**User**	**Number of robotic systems (*n* = 71)**	**in percent %**
Persons in need of care	n = 34	48%
Caregivers	n = 24	34%
Other professional groups	n = 7	10%
Relatives	n = 6	8%

### Possible applications of assistive robotic systems in nursing care

Assistive robotic systems can be divided into the following six fields of applications: Information and patient data processing, assistance with activities of daily living, fetch and bring activities, telepresence and communication, monitoring, safety and navigation, and complex assistance systems.

#### Information and patient data processing

With the help of the assistive robotic systems (Table 3) in Song et al. [23] and Ahn et al. [24], health data and patient information are entered into a robotic system upon admission to a hospital. Individual task plans are created and the robotic system navigates the patients to the appropriate departments, including diagnostic departments, using a follow-me function. The goal is to channel requests directly upon entering the hospital and facilitate the input and processing of patient information [23, 24], but also to provide directions for visitors and patients [25]. The Terapio robot was developed for medical or nursing rounds. The robot accompanies staff during walk-throughs or rounds and enables patient data entry and progress display [26]. In Stoevesandt et al. [27], the Pepper robot was used to educate patients about magnetic resonance imaging. In addition, assistive robotic systems are used as a communication channel [28], for example in case of an emergency [29].

**Table 3:** Assistive robotic systems for information and patient data processing (own representation)

**Author**	**Acronym: Function**	**State of development**	**Setting**	**User**
Pranathi et al., 2020 [29]	-: Message in case of emergency to caregivers	Development	Clinical care	Caregivers Persons in need of care
Tasaki et al., 2015 [26]	Terapio: Entering patient data			Caregivers Medical staff
Narayanan et al., 2022 [28]	-: Communication of information to patients	Test phase lab		Caregivers Persons in need of care
Song et al., 2017 [23]	-: Entering patient data			Persons in need of care
Stoevesandt et al., 2021 [27]	Pepper: Diagnostic information			Persons in need of care
Ahn et al., 2015 [24]	CareBot, ReceptonistBot, RoboGen: Entering patient data	Test phase field		Caregivers Persons in need of care relatives
Ma et al., 2021 [25]	-: Information/ directions on entering hospital			Caregivers Persons in need of care

#### Assistance with activities of daily living

Assistive robotic systems to support activities of daily living (Table 4) were developed for groups of people with special care needs. Their aim is to make everyday life easier and to maintain the independence of those affected. For this purpose, the technical systems provide step-by-step instructions or provide objectes [14, 30–32]. The robots Ed [31, 32] and iRobot [14] were developed to provide audiovisual instructions on how to perform specific activities. The Ed robot was developed for people with dementia or mild cognitive impairment. It guides users to make tea and wash their hands [31, 32]. The NAO robot assists people with neuromuscular diseases at home by reaching for objects and providing them to the person [30].

**Table 4:** Assistive robotic systems to support activities of daily living (own representation)

**Author**	**Acronym: Function**	**State of development**	**Setting**	**User**
Zhang et al., 2021 [30]	NAO: reaching/ providing objects	Test phase lab	Home care	Persons in need of care
Begum et al., 2013 [31] Wang et al., 2017 [32]	Ed: audio-visual instructions			Persons in need of care Relatives
Buhtz et al., 2018 [14]	iRobot: instructions for making tea/ washing hands			

#### Fetch and bring activities

The assistive robotic systems for pick-up and delivery activities could be divided into the following four sub-areas: Assistive systems for food and beverage service, for medication delivery, for contact reduction in infections, and user-related delivery services.

#### Food and beverage service

Assistive robotic systems for food and beverage service (Table 5) deliver meals and beverages directly to patients [29, 33–35]. The i-merc robotic system enables delivery of special dietary foods after input from dietitian staff [34]. As a robot for inpatient long-term care, Care-o-Bot 4 serves small snacks and drinks to residents [35].

**Table 5:** Assistive robotic systems for fetch and carry activities: food and beverage (own representation)

	**Author**	**Acronym: Function**	**State of development**	**Setting**	**User**
Food and beverage	Pranathi et al., 2020 [29]	-: food and beverage	Development	Clinical care	Caregivers Persons in need of care
	Haider et al., 2020 [33]	-: food and beverage			
	Carreira et al., 2006 [34]	i-merc: food transport with heating system, special dietary food			Caregivers Service staff Dietary assistance
	Graf, 2019 [35]	Care-o-Bot 4: snacks and drinks	Test phase field	Long term care	Persons in need of care Caregivers

#### Medication delivery services

Some assistive robotic systems deliver medications directly to patients (Table 6) [28, 29, 33, 36–38]. In this context, the robot in Haider et al. [33] is directly linked to the hospital pharmacy and delivers the ordered medication to the patients from there. The robot TUG is also stocked by the pharmaceutical staff in the pharmacy, but this robot delivers the order to the ward staff [39–41]. The WDBOT robotic system includes the complete ordering and delivery process: the required medications are ordered by the nursing staff at the corresponding ward. The robot collects the medications and is distributed to the patients together with the nursing specialist. For safety reasons, the nurse checks the medication and confirms the dispensing on a tablet [42]. Another system was developed for use in an oncology department. Its purpose was to transport radioactive materials [43].

**Table 6:** Assistive robotic systems for fetch and carry activities: medication delivery (own representation)

	**Author**	**Acronym: Function**	**State of development**	**Setting**	**User**
Medication delivery	Pranathi et al., 2020 [29]	-: medication delivery	Development	Clinical care	Caregivers Persons in need of care
	Haider et al., 2020 [33]	-: medication delivery			
	Hasan et al., 2020 [36]	-: bedside delivery, reminder			
	Antony et al., 2020 [37]	-: delivery to patient			
	Chien et al., 2019 [38]	-: delivery to patient when they are on the way			Caregivers Persons in need of care Relatives
	Dasanayake et al., 2018 [42]	WDBOT: ordering via software and delivery with robot	Test phase field		Caregivers Persons in need of care Medical staff Service staff
	Narayanan et al., 2022 [28]	-: delivery to patient	Test phase lab		Caregivers Persons in need of care
	Dallal et al., 2012 [43]	-: Transport of radioactive materials			Caregivers Persons in need of care Laboratory staff Medical Staff
	Kirschling et al., 2009 [39] Mutlu & Forlizzi, 2008 [40] Summerfield et al., 2011 [41]	TUG: stocking in pharmacy, autonomous delivery to caregivers	Use in practice		Caregivers Pharmacy staff Service staff Domestic staff

#### Contact reduction for infections

The goal of these assistive robotic systems is to minimize physical contact between patients and staff, both to prevent infection and to reduce the workload of health care professionals. In light of current events, the systems were developed and tested for the clinical care of COVID-19 patients (Table 7). The automated systems are designed to deliver drinks, meals, or medicines to infected individuals [44–47]. A system was developed to deliver materials to caregivers in an isolation room [45, 46].

**Table 7:** Assistive robotic systems for fetch and carry activities: contact reduction (own representation)

	**Author**	**Acronym: Function**	**State of development**	**Setting**	**User**
Contact reduction	Rusdi et al., 2021 [44]	-: supply of goods, COVID-19	Development	Clinical care	Persons in need of care
	Thamrongaphichartkul et al., 2020 [45] Vongbunyong et al., 2020 [46]	-: delivery of food or medical/ nursing materials to persons in isolation, COVID-19	Development Test phase field		
	Dadi et al., 2021 [47]	-: delivery of food, drinks, medication, COVID-19	Test phase lab		Caregivers Persons in need of care

#### User- related delivery services

In addition to delivering food, beverages, or medications, assistive robotic systems are also used to deliver and transport laboratory materials, documents and further undefined objects (Table 8) [48–53]. The intelligent nursing trolley can be navigated by caregivers via a smartphone and delivers wound materials (hospital) or laundry (inpatient long-term care) to the destination, depending on the application. The goal is to reduce walking distances, thereby reducing the burden on nursing staff [35, 55–56]. The CASERO robot transports clean laundry to the station and takes the dirty laundry with it [10, 57, 58]

**Table 8:** Assistive robotic systems for fetch and carry activities: user-related delivery services (own representation)

	**Author**	**Acronym: Function**	**State of development**	**Setting**	**User**
User-related delivery	Ettelt et al., 1998 [48]	ROMAN: laboratory materials, medication, documents	Development	Clinical Care	Caregivers
	Abubakar et al., 2020 [49] Saadatzi et al., 2020 [50]	ARNA: non-defined objects	Development Test phase field		Caregivers Persons in need of care
	Yamamoto et al., 2019 [51]	-: Identifying and picking up objects with a gripper arm	Test phase field	Home Care	Persons in need of care Relatives
	Graf et al., 2013 [10] Graf & Jacobs, 2011 [57] Graf et al., 2012 [58]	CASERO: Supply and removal of laundry		Long term care	Caregivers Service staff Domestic staff
	Graf, 2019 [35] Graf et al., 2016 [54] Graf, 2018 [55] Schiller et al., 2019 [56]	Intelligent nursing trolley: Operation via mobile device, supplies wound material or laundry utensils (depending on setting)		Clinical care Long term care	Persons in need of care Caregivers
	Früh et al., 2018 [52]	LIO: picking up and bringing, non-defined objects	-	Clinical,- Long term,- Home care	Caregivers Persons in need of care
	Klein et al., 2018 [53]	SCITOS: non-defined objects	-	Home care	Persons in need of care

#### Telepresence and communication

A key feature of the systems described in Table 9 is being able to interact with people. Ienca et al. [59] describe a growing share of assistive technologies for people with dementia or cognitive impairment, which focus on social-assistive functions or telemedicine. The Giraff robot is an example of this [14, 59]. Assistive robotic systems for this application area enable robotic communication between health care professionals, caregivers, relatives and patients. For example, communication is possible via speech recognition or via input into a tablet [43, 52, 60]. The IVO & Tommy system allows communication between COVID-19 patients and caregivers. Communication was enabled by audio and touch screen functions. The aim was to protect staff from infection by reducing contact [60].

**Table 9:** Assistive robotic systems for telepresence and communication (own representation)

**Author**	**Acronym: Funtion**	**State of development**	**Setting**	**User**
Buhtz et al., 2018 [14] Ienca et al., 2017 [59]	Giraff: communication between persons in need of care and relatives	Test phase Use in practice	Home care	Persons in need of care Relatives
Dallal et al., 2012 [43]	-: Telepresence robotics, communication between caregivers and persons in need of care	Test phase lab	Clinical care	Caregivers Persons in need of care Laboratory staff Medical Staff
Bartosiak et al., 2022 [60]	IVO & Tommy: robotic communication between healthcare workers and patients through the use of audio and touch screen functions, COVID-19	Use in practice		Caregivers Persons in need of care
Früh et al., 2018 [52]	P-Care: verbal communication via voice recognition		-	

#### Monitoring, safety and navigation

One function of this category (Table 10) is the monitoring and documentation of patients' vital signs. The IVO & Tommy robot also has the function of reading vital signs in the patients' room via an integrated camera, where, on the other hand, the Silbot robot enables daily recording of general condition and mental state [37, 53, 60, 61]. Another safety aspect is the reminder function, such as medication reminders [53, 61, 62]. The robotic system of Arthanat et al. [63] is also capable of sending alerts to predefined individuals, such as family members, if medications have not been taken. In addition to medication reminders, the Pearl robot also handles personal hygiene reminders and meal and drink reminders [64].

**Table 10:** Assistive robotic systems for monitoring, security and navigation (own representation)

**Author**	**Acronym: Function**	**State of development**	Setting	**User**
Mahajan & Vidhyapathi, 2017 [62]	-: monitoring of well-being and orientation, information in case of changes, monitoring and alarm in case of falls, transmission of the situation via camera	Development	Clinical care	Persons in need of care
Abubakar et al., 2020 [49] Saadatzi et al., 2020 [50]	ARNA: patient escort service, robot as walking assistance			Caregivers Persons in need of care
Antony et al., 2020 [37]	-: Measurement of vital parameters, transmission to staff			
Chien et al., 2019 [38]	-: Carrying heavy loads by follow-me function, navigation		Clinical care Long term care	Caregivers Persons in need of care Relatives
Arthanat et al., 2020 [63]	-: reminder and accompanying function for taking medication, warning when leaving the home, reminder after a certain time, call from emergency contacts	Test phase lab	Home care	Persons in need of care Relatives
Pineau et al., 2003 [64]	Pearl: Remembering daily activities (medication, eating, drinking, personal hygiene, appointments), observing activity, module includes knowledge about person, navigating through environment, follow-me function		Long term care	Caregivers Persons in need of care
Song et al., 2017 [23]	-: navigation to units (e.g. diagnostics)		Clinical care	Persons in need of care
Bartosiak et al., 2022 [60]	IVO & Tommy: monitoring of vital parameters by camera on the robot	Use in practice		Caregivers Persons in need of care
Law et al., 2019 [61]	Silbot: alarm function, reading out the daily schedule, medication reminder, sending emergency calls, monitoring (daily recording of general condition and mood)	Use in practice	Long term care Home care	Persons in need of care
Klein et al., 2018 [53]	SCITOS: reminder function, environment monitoring, fall detection and help call system	-	Home care	Persons in need of care

The function of navigation belongs to this category, as these systems support the safe mobility of individuals. The ARNA robot accompanies patients. It serves as a walking aid and thus provides stability [49, 50]. The robotic system at Mahajan & Vidhyapathi [62] additionally performs lifting and carrying of objects, such as oxygen equipment, IVs, and drains. The target users for this system is individuals in acute inpatient settings during the postoperative period. For orientation in hospitals, robots also serve as a mobile navigation system using follow-me function [23, 38].

#### Complex assistance systems

Complex assistive robotic systems for assistance (Table 11) perform multiple tasks in the areas of service, communication, safety, social participation and employment. The Kompaii and RAMCIP robots assist individuals by providing information and communication functions. Kompaii can send an emergency call to a control center or remind people to take medication. RAMCIP is additionally able to record fall events. In addition to entertainment functions, such as music playback function, game function and weather service applications, there is the possibility to use video telephony. In some cases, the systems also move autonomously in space, detect obstacles, and can deliver light items such as medication or water to the person [53, 65–67]. The Care-o-Bot 3 robot was specifically designed for inpatient long-term care and home settings. It performs simple pick-up and drop-off services, serves drinks, speaks directly to residents, and provides entertainment through memory games, music, and poetry. In the home setting, it is possible to interface with an emergency call center, which can, for example, send an emergency call in case of a fall event [10, 53, 57–58].

**Table 11:** Complex assistive robotic systems for assistance tasks (own representation)

**Author**	**Acronym: Function**	**State of development**	**Setting**	**User**
Klein et al., 2018 [53] Wu et al., 2014 [65] Zsiga et al., 2018 [66]	Kompaii: information service (weather, schedule, time), medication reminder, socioassociative functions (video telephony, games, music), safety and health (emergency call signal, health check report), navigation in rooms, obstacle detection, carrying light objects	Use in practice Test phase lab Test phase field	Home care	Persons in need of care
Gerlowska et al., 2018 [67]	RAMCIP: fetch and carry (e.g. medication, water), support and monitoring of taking medication or cooking, detection of impending fall situation, social interaction/ communication function	Test phase lab		
Graf & Jacobs, 2011 [57] Graf et al., 2012 [58] Graf et al., 2013 [10] Klein et al., 2018 [53] Schiller et al., 2019 [56]	Care-o-Bot 3: drink and snack service, automated dispensing on tray, entertainment through memory games (music, poems), grasping objects (opening door)	Test phase field	Long term care Home care	Persons in need of care Caregivers

### Social and nursing science findings

The second aim of the review was to describe an overview of existing social and nursing science findings. The included publications were analyzed with regard to common research foci and summarized by content analysis. The descriptive description of the study findings is divided into *integration and impact on practice* and *attitude and acceptance of older people towards assistive robotic systems*.

#### Integration of technology and impact on practice

Results were summarized from four studies. Two of the studies followed a qualitative research approach and one study each followed a quantitative and mixed-method approach.

Mutlu & Forlizzi [40] used a qualitative ethnographic research design to explore the effects and changes on organizational and social-communicative factors during the use of an autonomous robot for fetch and delivery activities in an acute internal medicine ward and an obstetrics unit. The results, analyzed according to the principles of grounded theory, were able to describe differences in terms of perceived benefits, acceptance, interactions, and work-organizational changes. The differences relate in particular to the integration of the robot into the workflow and to the perception and interaction with the technical system. On the acute internal medicine ward, the robot was hardly tolerated. The integration into work processes and the resulting interruptions within the daily work routine were perceived as disturbing. In contrast, the robot could be integrated into work processes on the obstetrics ward. In the study by Summerfield et al. [41] similar results are described. Here, the use of a medication delivery robot was evaluated in an intensive care unit. The change in the medication delivery process contributed to additional activities being transferred to nurses. This contributed to decreased acceptance toward the technical system. Another problem was the technical failures of the robot, which led to further delays in the workflow and additional deliveries. The study by Arthanat et al. [63] also indicated that acceptance is based on technical aspects, such as navigability, humanoid characteristics, user interface, and adaptability. The aim of the study was to test an assistance robot with a group of relative caregivers and to collect their views on a possible integration into a home setting. The technical problems also led to a rejection attitude. Other reasons for rejection were the technical complexity and the failure to develop assistive robotic systems with a hands-on and person-oriented approach.

Furthermore, in Mutlu & Forlizzi [40] the culture-specific and the social-emotional context play a role. Due to the disease spectrum of the patients in the internal medicine ward, a special relationship between nurses and their patients could be observed. Therefore, in conjunction with a work environment characterized by stress and noise, deliveries and the attention they required were perceived as disruptive to the robot. Negative attitudes extended to physically and verbally assaulting the robot. In contrast, the nurses in the obstetrics ward described the relationship with the robot as positive, helpful, and friendly. Due to patient transports or emergencies, the internal medicine department often had a stressful work environment. Objects standing around also presented obstacles for the robot and led to collisions with staff, patients or visitors. Such incidents triggered dissatisfaction towards the robot, but this was less frequently recorded on the obstetrics ward. Regardless of the focus of care, respondents rated it critically when the robot stopped in the corridors for several minutes. This caused anxiety among nurses, as the robot was seen as a disruptive factor in the event of an emergency. Complementing this, Kirschling et al. [39] found in their study that such technical and situational difficulties reduced the utilization of the robotic system.

#### Attitude and acceptance of elderly people towards assisitve robotic systems

Results were summarized from two studies. One study followed a qualitative approach and the other a mixed method approach. Acceptance of and attitudes toward assistive robotic systems in home care can be described as heterogeneous. Wang et al. [32] investigated the attitudes of older people with a mild form of Alzheimer's disease and their relatives towards a assistive robotic system to support activities of daily living in the home setting. From the semi-structured interviews, different perspectives on the use can be described. A part of the elderly persons and their relatives rejected the robotic system in principle, while other persons were open to the idea but did not see a current personal need for such technological support. The negative attitude was justified from different perspectives. On the one hand, the participants did not see it as necessary, since home care was already provided by their relatives. On the other hand, the associated cost factor and the desire for human care represented a hurdle. Also, the elderly respondents from the study by Wu et al. [65] noted that they could not demonstrate an individual need for a robotic system. Rather, they described a threat of dependency from the use of robotic technologies. The resulting stigma combined with the desire for independence within care had a negative impact on acceptance. Furthermore, the potential of loneliness and dehumanization was described. There was a risk that contact with relatives would be restricted and that interpersonal relationships could suffer as a result. In contrast, Wang et al. [32] acknowledged that the use of assistive robotic systems could reduce worry, anxiety and stress, which could lead to a better relationship between relatives and care recipients. The assessment of the role that an assistive robotic system could take in such a care constellation ranges from a friend that supports social and emotional needs to a machine that enables the execution of household activities and offers little scope for personal relationships. The older persons interviewed assess the robot as a measure against social isolation and note that contact with a machine can never replace that with a human being. Building a relationship is seen as potentially possible, however, according to the interviewees, it will never be like the relationship with a human. For some older people, it was important to learn how to use new technologies so as not to become alienated from modern society. Other factors influencing acceptance were age and existing technology skills. The family caregivers had a positive attitude towards the use of assistive robotic systems. Interestingly, they assumed that the persons in need of care were also positively disposed towards the use [32, 65].

## Discussion

The results of this systematic literature review show that assistive robotic systems in nursing care perform a variety of tasks in a wide range of settings. From the 47 included publications, 39 different systems could be identified. Almost one third of the systems were in the development phase and more than half in the test phase. Only 7 systems were already in use in nursing practice at the time of the search. With regard to the settings of use, it became apparent that more than half of the systems were developed for use in clinical care. The focus of the users is on persons in need of care and nursing professionals. Relatives, who often take over essential responsibilities in the care of persons in need of care, still play a subordinate role in this research field.

### Assistive robotic system—a conceptual approach

A look at the literature shows that there are different categories and definitions of robotic systems in nursing. In their systematic literature review, Maalouf et al. [8] subdivide robotic systems into the categories "assistive robots" and "social assistive robots." The category "assistive robots" includes technical systems such as service, transport and surveillance robots. Robots that support food intake and personal hygiene are also classified here. Some of the application areas from the present literature research can also be classified as "assistive robots", e.g. the assumption of service activities or the possibility of monitoring. However, although both papers deal with "assistive" systems in nursing, there are still differences. For example Maalouf et al. [8] also address areas of application that involve direct and near-patient activities, such as personal care, mobility, and food intake. Considering the term "care robots", according to Hülsken-Giesler & Daxberger [68] there is no unified definition for "robots" and according to Bendel [68] therapy-related robots are close relatives and robots for sexual needs are distant acquaintances of care robots. The discussion illustrates that there are different definitions of terms and categories existing in this research field. For the concept of "assistive robotic systems for care", a definitional attempt is provided for discussion. The concept of "assistive robotic systems for care" has a clear reference to persons in need of care or persons who are integrated in direct nursing care. The aim is to support care through simple, action-oriented tasks and to promote direct person-related contact. This definition distinguishes "assistive robotic systems for care" from therapy-related systems or systems for direct patient-centered care actions.

### A question of acceptance

The evaluation of the scientific evidence showed that social science studies are largely concerned with issues of acceptance toward assistive robotic systems, in addition to the question of integration into practice. The studies show that acceptance is related to the individually perceived need for care. On the one hand, assistive robotic systems may increase loneliness and dehumanization. On the other hand, it has been noted that assistive robotic systems can provide relief within care situations [32, 65]. The review paper by Peek et al. [70] explored the question of what factors influence acceptance towards technology. Again, concerns about technology, such as high cost, inefficiency, and stigma could be located. But perceived need and benefit are also influencing factors. It can be concluded that caregivers, product developers, and policy makers face a variety of factors that influence adoption. Therefore, it is necessary to integrate all stakeholders involved in the process and, in particular, persons in need of care and their relatives into the development and implementation process. Furthermore, findings on technology acceptance need to be related to issues of implementation and ethics.

### Limitation

In the publications, some of the robotic systems were described briefly and exemplarily, others in great detail. Therefore it was not always possible to guarantee a detailed description of the technical systems and their possible applications in the results section. The definition of assistive robotic system chosen at the outset may have led to technical systems and relevant study results being disregarded. Despite the extensive and supplementary database research, it must be acknowledged that relevant sources of information were overlooked, as the research was only conducted in German and English. The heterogeneous and partly qualitatively poor studies focused for the most part only on technical and application-related questions, so that nursing science and care-related issues could hardly be addressed.

## Conclusion

The results illustrate that the transfer of assistive robotic systems into nursing care is still in its beginnings. Nursing professionals and people in need of care are already involved in the development and testing of assistive robotic systems. This participation has to take place again in the case of implementation in a real practice environment. The literature review clarifies that relatives are hardly involved in technology development and testing. However, they are an important part of home care and should be more integrated into technology development and nursing science. This can be realized by co-creative and participatory research approaches. In this context, qualitative and especially ethnographic methods prove their worth, because in interdisciplinary projects, mutual understanding and empathy for the other party is a basic requirement for technology development and practice transfer. The identified applications for assistive robotic systems are diverse and targeted to different settings and groups of people. The assistive robotic systems are often developed to take over a highly specialized activity. This fact reduces the fear that in the near future nursing professionals and complex nursing activities can be replaced by assistive robotic systems. However, they have an impact on nursing care situations, which is why ethical and social issues must be taken into account during development. There is also a need for research to determine the impact on nursing practice, as well as facilitating and inhibiting factors for implementation. To achieve this, projects must be evaluated and become the subject of research. In addition, technology development and transfer must be discussed at the political and legal level, because the transfer of technical systems to standard care is only possible with their support.

## Data Availability

All data are available from the corresponding author upon reasonable request.

## Abbreviations

CINAHL: Cumulative Index to Nursing and Allied Health Literature
PRISMA: Preferred reporting items for Systematic reviews and Meta-Analyses
JBI: Joanna Briggs Institute
PCC: Population, Context, Concept
QCRI: Qatar Computing Research Institute
